# Case of Phlegmonous Erysipelas

**Published:** 1884-04-20

**Authors:** T. M. Beaty

**Affiliations:** Okolona, Miss.


### CASE OF PHLEGMONOUS ERYSIPELAS.

BY T. M. BEATY, OKOLONA, MISS.

There are few diseases which have been, or could be, subject to more varied methods of treatment than Erysipelas. Before proceeding to discuss this case, I would say that I have been a subscriber to this popular journal for nearly two years, and have seen a great many diseases discussed at length. The only apology I can offer for troubling you with this case is that I would like to induce the members of the profession to give their views upon this subject.

I will try and present, briefly, a case in practice. I was called to see Annie F——. She was taken suddenly during the night, a week or more prior to my visit, with inflammation of the arm. The inflammation extended deeply into the different tissues, the skin, muscular and cellular substance. As it progressed, suppuration took place and destroyed large portions of the cellular and adipose tissues, above and below the elbow joint.

I am confident the sloughing was caused from high tension of the parts, produced by infiltration of serosity.

I first removed all sloughs; used for local application a solution of tincture of iodine and carbolic acid; left a purgative medicine, directed to be given until the bowels were thoroughly evacuated; prescribed belladonna, three drops of the fluid extract every hour.

The reasons that actuated me to try belladonna are, that under moderate stimulant doses the visible number of capillary vessels is increased; but, at the same time, the individual vessels undergo such a contraction that the amount of blood in the part is diminished instead of being increased. It is also claimed that its hypnotic effects are produced by the contraction of the vesels of the brain. It is impossible to give a clear and consistent rationale of the mode of action of belladonna; for it is like all other medicines which act directly through the nervous system. Small and large doses produce opposite effects. I found belladonna to be singularly efficacious in the relief of this most distressing disorder.

Now, gentlemen, this is a frequent disease and is very painful; if suppuration occur it is prolonged.

I have called your attention to it solely with regard to its therapeutics. It presents no difficulty in diagnosis, though very obscure in its pathology. In severe cases I believe that the treatment followed in this patient will give very satisfactory results. Therefore, I don't think the professional brethren ought to condemn it without subjecting it to a trial.

